# Modelling Oscillatory Patterns in the Bovine Estrous Cycle with Boolean Delay Equations

**DOI:** 10.1007/s11538-021-00942-z

**Published:** 2021-11-02

**Authors:** Mascha Berg, Julia Plöntzke, Heike Siebert, Susanna Röblitz

**Affiliations:** 1 Zuse Institute Berlin, Berlin, Germany; 2 Department of Mathematics and Computer Science, Freie Universität, Berlin, Germany; 3 Computational Biology Unit, Department of Informatics, University of Bergen, Bergen, Norway

**Keywords:** BDEs, Bovine estrous cycle, Hormones, Dynamical systems, Semi-discrete models, Boolean delay equation

## Abstract

Boolean delay equations (BDEs), with their relatively simple and intuitive mode of modelling, have been used in many research areas including, for example, climate dynamics and earthquake propagation. Their application to biological systems has been scarce and limited to the molecular level. Here, we derive and present two BDE models. One is directly derived from a previously published ordinary differential equation (ODE) model for the bovine estrous cycle, whereas the second model includes a modification of a particular biological mechanism. We not only compare the simulation results from the BDE models with the trajectories of the ODE model, but also validate the BDE models with two additional numerical experiments. One experiment induces a switch in the oscillatory pattern upon changes in the model parameters, and the other simulates the administration of a hormone that is known to shift the estrous cycle in time. The models presented here are the first BDE models for hormonal oscillators, and the first BDE models for drug administration. Even though automatic parameter estimation still remains challenging, our results support the role of BDEs as a framework for the systematic modelling of complex biological oscillators.

**Supplementary Information:**

The online version supplementary material available at 10.1007/s11538-021-00942-z.

## Introduction

There exist different approaches to create mathematical models of biological systems. Two commonly used modelling types are ordinary differential equations (ODEs), with continuous time and continuous values for the model components, i.e. “continuous space”, and Boolean models, with discrete time and discrete space. Both types of models have their advantages and disadvantages. They need different knowledge of the modelled system and result in different types of information. Developing an ODE model that adequately reflects the behaviour of a particular biological system requires detailed knowledge of the underlying mechanisms. Moreover, accurate quantitative values for the model parameters or sufficient data to determine the parameter values using some suitable parameter estimation method are needed. The construction of a Boolean model, on the other hand, requires only qualitative knowledge about a system, for example, the type of interaction (inhibitory or stimulatory) between two species in the model. However, Boolean models cannot adequately reflect time scales.^1^ Hence, it is evident that exploring new types of models that combine the properties of ODEs and Boolean models is a promising approach. There exist various modelling frameworks with discrete time and continuous space as, for instance, difference equations, or with continuous time and discrete space. One approach for the latter, presented by Stoll et al. (2012), uses continuous-time Markov chains. Another example are Boolean delay equations (BDEs). In BDEs, the components are linked with logical functions. A delay matrix ensures that the components do not influence each other immediately, but after a certain time. The delays enable the modelling of different time scales in processes such as signalling or gene transcription.

Even if there already exists an ODE or Boolean model for a particular biological system, it can be elucidating to create a model of another type, such as a BDE, for that system. An analysis of the additional model can either be performed independently from the existing models or it can be carried out in comparison with the existing models (Stötzel et al. 2015). ODE models have the advantage that they are very detailed, meaning that they provide an exact time course of species with high resolution. Their disadvantage is that very precise knowledge of the system is required to build and parameterise the model, and that global analysis is often impossible due to model complexity. In contrast, BDEs can be analysed more systematically due to their finite state space. Unlike Boolean models, BDEs can incorporate more precise time scales, which allows for the simulation of external stimuli with delayed effect, e.g. drug administration.

The aim of our work is to analyse the potential of BDEs for modelling a periodic, biological system by creating a BDE model for the bovine estrous cycle. The starting points are an existing ODE model (Stötzel et al. 2014a) and a Boolean model (Stötzel et al. 2015) that was derived from the same ODE model. The BDE models presented here are in fact the first BDE models for a hormonal system. Since BDEs always become periodic for rational delays, they are periodic in numerical simulations after a certain time, the so-called transient. This intrinsic periodicity suggests that BDEs might be a suitable modelling approach for the hormonal cycle.

In this paper, we introduce two BDE models, which we refer to as *Model A* and *Model B*, whose logical functions are based on the logical functions derived in Stötzel et al. (2015). *Model A* is derived directly from the existing ODE and Boolean model, whereas *Model B* is based on the same equations but includes a modification of a particular biological mechanism to incorporate biological knowledge.

If other models are already available for a certain system, one possible approach is to use these models and to replicate their trajectories with the new model, as we did for *Model A*. Possible shortcomings of the existing models may, however, be adopted. Therefore, we also test the alternative approach of creating a model based on biological knowledge, which results in *Model B*. In order to compare the two models, we validate them with two in silico experiments. One experiment induces a switch in the oscillatory pattern upon changes in the model parameters. The other experiment simulates the administration of a hormone that is known to shift the estrous cycle in time.

The potential use of these models for further research lies in the field of experimental design, as well as in testing treatment protocols in silico, thereby contributing to the 3Rs: replacement, reduction, and refinement of animals in testing.

## Methods

### Boolean Delay Equations

Boolean delay equations are continuous-time models over a Boolean state space. BDEs are categorised as semi-discrete models (Ghil et al. 2008). In that manner, BDEs can be placed between Boolean models (with discrete time and discrete space) and ODEs (with continuous time and continuous space).

BDEs were first introduced by Ghil and Mullhaupt (1985). They have been applied to a wide range of research areas, comprising inter alia the following: climate dynamics (El Niño/Southern Oscillation (ENSO) phenomenon (Saunders and Ghil 2001)) and climatic systems (Ghil et al. 1987), damage propagation in networks (Coluzzi et al. 2011; Colon and Ghil 2017) earthquake modelling (Zaliapin et al. 2003), genomic interactions (Oktem et al. 2004), and circadian systems (Akman et al. 2012). To our knowledge, the herein presented approach is not only the first application of BDEs to a hormonal regulatory system, but also the first BDE modelling approach for a biological oscillator and for the effect of drug administration.

The main advantage of modelling with BDEs compared to ODEs is that it affords a very simple and intuitive way of model construction. Modelling with ODEs either needs deep knowledge about underlying mechanisms or appropriate ansatzes to represent them. Even if parameter values do not need to have obvious biological correlates, measurement data are needed to numerically estimate their values. In contrast, BDE models involve fewer parameters and usually need less information of the underlying mechanisms. For each species, one needs to specify a logical function, and for each tuple of species connected by a logical function, one needs an entry in a delay matrix which represents the time span it takes until the influence takes place. In terms of optimisation, the use of BDEs reduces the number of parameters drastically compared to ODEs. Since the number of possible logical configurations is finite, a systematic search for the model that best matches the data is possible (Doherty et al. 2017).

Regarding initial values, BDEs need more information than ODEs. To conduct a simulation with ODEs, one needs to assign one value for each species at the starting time of the simulation. For BDEs, one has to define a time series with the length of the largest delay (usually scaled to 1) for all species. Then, the solution of the BDE system exists for arbitrary time intervals and is unique; a proof of this can be found in Ghil et al. (2008). In this *initial period*, expert knowledge about the biological system is incorporated. In many cases, the solution is sensitive to the initial period, which means that even small changes in the initial period can lead to very different periodic behaviours, whereby not all of these solutions are biologically reasonable.

#### Formal Definition of BDEs

According to Ghil et al. (2008), BDEs can be formally defined as follows:

*A BDE system consists of n species* $x_1,\dots ,x_n \in \{0,1\}$, *corresponding to the species being either ON or OFF, or being below or above a certain threshold. The time-dependent value of each species is given by a logical function:*

1 $$\begin{aligned} x_i(t)=f_i\big (x_1(t-\theta _{i,1}),\dots ,x_n(t-\theta _{i,n})\big ) \end{aligned}$$

*for* $i=1,\dots ,n$, *where* $f_i:\{0,1\}^n \rightarrow \{0,1\}$ *are Boolean functions and* $\theta _{i,j}$ *are delay values, summarised in the delay matrix:*

$$\begin{aligned} \varTheta = \left( \begin{array}{ccc} \theta _{1,1} &{} \cdots &{} \theta _{1,n} \\ \vdots &{} \ddots &{} \vdots \\ \theta _{n,1} &{} \cdots &{} \theta _{n,n} \end{array}\right) . \end{aligned}$$

*The system is normalised so that the largest delay is 1. *

As by Ghil et al. (2008), only autonomous BDEs are considered that means without explicit time dependence of the logical functions. Each species’ value at time *t* depends on the values of other species and/or its own value at an earlier time point, defined by the delays.

#### Properties of BDEs

From a theoretical point of view, BDEs have some very interesting properties. One is described by the so-called pigeon-hole lemma (Ghil et al. 2008), which states that all BDEs with a delay matrix containing only rational delays, become periodic after a certain amount of time. Since numerical simulations of BDEs can always handle just rational delays (because of machine accuracy), all numerical simulations lead to BDEs that after some (perhaps very long) time reach a periodic orbit (Ghil et al. 2008) or a stable steady state (meaning constant values of all species). Since biological systems usually reach stable behaviour, this property does not restrict the class of biological systems that can be modelled with the BDE formalism. In particular, it is this property of periodicity that gave rise to the idea of modelling the hormonal cycle in terms of BDEs.

BDEs with instantly periodic solutions for all initial periods are called conservative, while all other BDEs are called dissipative. Dissipative BDEs might show transient behaviour before becoming periodic, at least for some initial data. Being conservative is equivalent to the property of being reversible, which means the time reversal of the system of BDEs is also a system of BDEs (Ghil et al. 2008).

From a theoretical point of view, dissipative BDEs have much more interesting behaviour. Relatively simple examples with irrational delays can be constructed in which the number of jumps on intervals of length 1 increases arbitrarily strongly with advancing time (Ghil et al. 2008).

Another surprising property of BDEs is called *periodic approximation* (Ghil et al. 2008), meaning that all solutions of a BDE system for a fixed finite time interval, even non-periodic ones, can be approximated by the periodic solutions of another BDE system with rational delays only. However, this other BDE might be intractable for interpretations with respect to application.

For a summary of more important theoretical results on BDEs, see Ghil et al. (2008).

### Previous Bovine Estrous Cycle Models

The bovine estrous cycle is the periodic cycle of changes in the levels of fertility related hormones and their influence on the body (for example growth of follicles) in female cattle. The basis of fertility is the regular production of an ovum that is released by a follicle. The remaining parts of the follicle transform into the corpus luteum (*CL*), which decays after a few days upon a certain hormonal signal. The part of the cycle during which the *CL* is present is called the luteal phase.

The model BovCycle is a mathematical model for the bovine estrous cycle, consisting of 12 ODEs and 54 parameters. The original BovCycle model was published by Boer et al. (2011b). It is capable of simulating the growth and regression of the bovine follicles and corpus luteum as well as the dynamics of the main fertility hormones during the estrous cycle.

In Stötzel et al. (2014a), a reduced model was published that is capable of reproducing the state trajectories of the original model. The reduced model contains ten ODEs and 38 parameters and was derived by model reduction techniques from the original BovCycle model. In both ODE models, the species are normalised such that their values are between 0 and 1.

In Stötzel et al. (2015), the reduced BovCycle model was used to introduce a comprehensible and systematic formalism to translate an ODE model into a Boolean model using a discrete transformation that resembles Euler’s method. The formalism for transferring the ODE to a Boolean model was described in a general framework so that it can be applied to any ODE model whose equations meet certain conditions (the system must be autonomous, and the right hand sides have to consist only of sums and products of monotone functions). It was shown by Stötzel et al. (2015) that the Boolean model reproduces the trajectories of the reduced BovCycle ODE model. In addition, some interesting global properties could be derived from the Boolean model that could be observed but not proven with the ODE model.

### Modelling Approaches

The results from Stötzel et al. (2015) demonstrate how a Boolean model allows for a systematic analysis of the system’s behaviour. However, for the simulation of certain experiments, such as drug administration, it is necessary to include time information in order to account for, e.g. different half-lives of drugs. BDEs meet exactly these requirements. It is known from experiments that the estrous cycle has a very stable cyclic behaviour. However, for some types of perturbation the system needs some time to converge back to the original cycle^2^. Therefore, building dissipative BDEs fits the desired behaviour of the model.

We present two different models for the bovine estrous cycle, *Model A* and *Model B*, which are discussed in detail in the following section. Both BDE models incorporate the same ten species as the reduced BovCycle model (Stötzel et al. 2014a), namely *CL*, *E*2, *FSH*, *Foll*, *GnRH*, *Inh*, *IOF*, *LH*, *P*4, $PGF2\alpha$. These abbreviations stand for the biological components specified in Table 1.

**Table 1:** Model components as described by Boer et al. (2011a) and Stötzel et al. (2012)

Model component	Explanation
*GnRH*	Gonadotropin releasing hormone
*LH*	Luteinizing hormone
*CL*	Corpus luteum
*P*4	Progesterone
$PGF2\alpha$	Prostaglandin F2$\alpha$
*IOF*	Inter-ovarian factors
*FSH*	Follicle stimulating hormone
*Foll*	Development of the total size of all follicles
*E*2	Estradiol
*Inh*	Inhibin

A very rough summary of the interaction of the species can be given as follows:

After ovulation, the remaining parts of the ovulated follicle transform into a mass of cells called the corpus luteum (*CL*). The hormone Estradiol (*E*2) is produced by the follicles, and Progesterone (*P*4) by the *CL*. Inhibin (*Inh*) is produced by dominant follicles and suppresses the follicle stimulating hormone (*FSH*). Gonadotropin releasing hormone (*GnRH*) is released by the *GnRH* pulse generator located in the hypothalamus, modulated by the hormones *P*4 and *E*2, depending on the stage of the cycle. *GnRH* causes the release of the gonadotropins luteinizing hormone (*LH*) and *FSH*, which stimulates the maturation of the follicles (*Foll*) and *LH* induces ovulation. The regression of the *CL* (called luteolysis) is caused by Prostaglandin F2$\alpha$ ($PGF2\alpha$), which is released when implantation does not take place. The inter-ovarian factors (*IOF*) are the summary of several components that control the effect of $PGF2\alpha$ on the *CL*. The growth of the follicles occurs in a wave-like pattern, with usually two or three waves per estrous cycle. Corresponding to that the hormone *FSH*, which stimulates the follicles’ growth, has in most cases two or three peaks per cycle. A more detailed description of the model components as well as the biological background can be found in Boer et al. (2011a) and Stötzel et al. (2012).

**Fig. 1:**
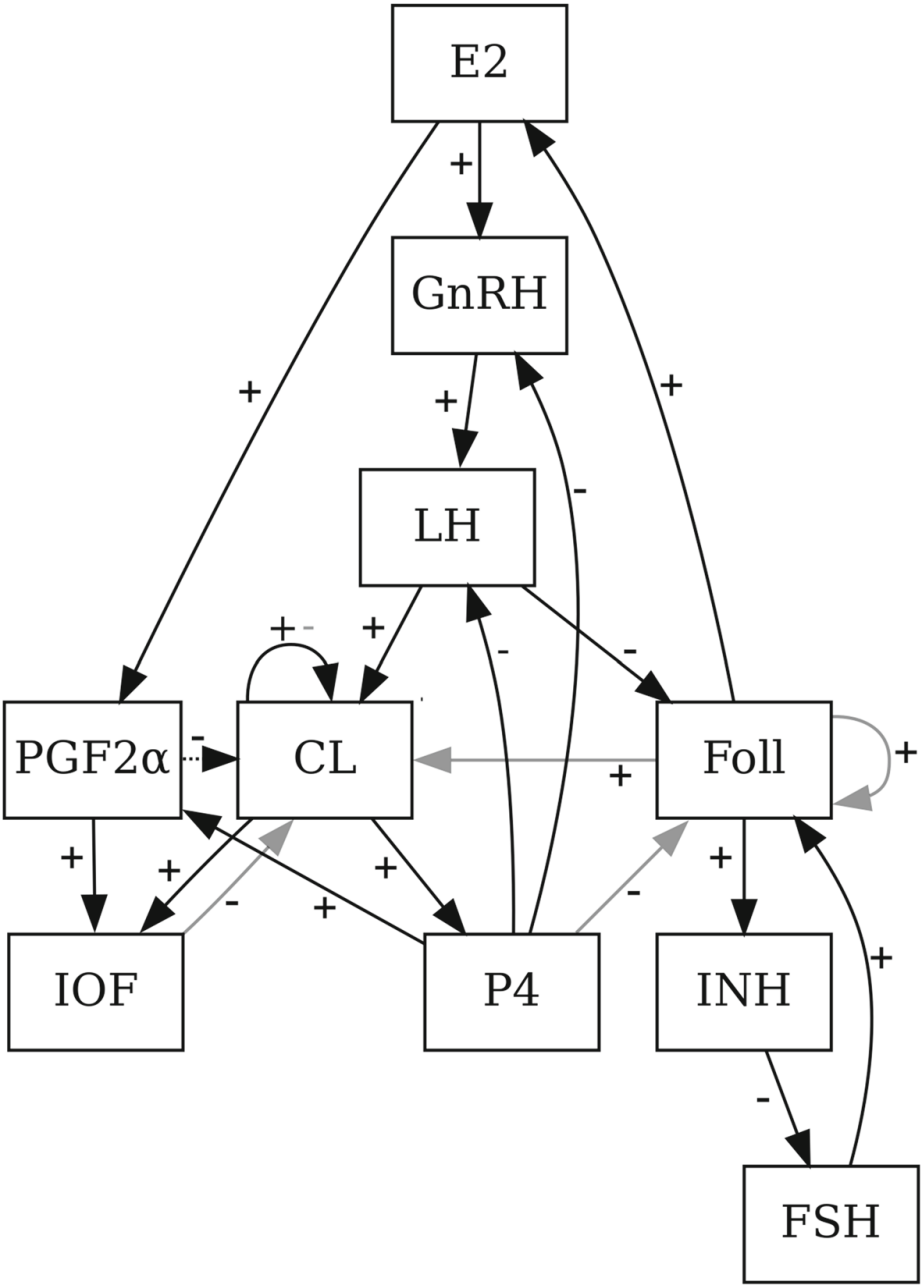
Interaction graph for both BDE models, *Model A* and *Model B*. Black solid lines stand for dependencies that exist in both models. Light grey colour indicates dependencies that exist in *Model A* but not in *Model B*. The dotted arrow line indicates a dependency which exists in *Model B* but not in *Model A*. The signs on the arrows mark whether the influence is stimulating or inhibiting

In Figure 1, the interaction graphs for both *Model A* and *Model B* are depicted to illustrate the dependencies between the model components. As basis for the two BDE models, the logical functions are derived from the truth tables of the Boolean model presented in Stötzel et al. (2015). These truth tables were found using the formalism described in Stötzel et al. (2015), where a Boolean update function is derived from an ODE system using a discrete transformation similar to Euler’s method.

## Results and Discussion

In the following, we present the two different BDE models as explained in the previous section. For some initial data, the two models show a transient, so they are dissipative. Since the main interest is in the periodic part of the solution, the initial periods are adjusted so that there is no transient left. Therefore, the property of being dissipative is not directly recognisable in the examples shown, but was recognised when the models were created.

### Model A

*Model A* was created to reproduce the trajectories of the reduced BovCycle model as accurately as possible. The resulting model is capable of simulating cycles with either two or three follicular waves.

#### Equations and Delays

All equations were extracted from the truth tables in Stötzel et al. (2015), except the equation for *Foll*. The equations of the BovCycle model are included as Online Resource 1.The original equation for *Foll* was:

$$\begin{aligned} Foll=\big ((FSH \vee \lnot P4) \wedge \lnot LH \wedge Foll \big ) \vee (FSH \wedge \lnot Foll ). \end{aligned}$$

Using this equation results in simulations with too many jumps in the *Foll* component, followed by too frequent jumps in all other components as well, which is not biologically reasonable. A figure provided as Online Resource 2 shows how a simulation looks like in this case. Through a systematic analysis of the simulation results it was possible to identify the term that caused the unwanted jumps. This term, $(FSH \wedge \lnot Foll)$, is an artefact from the ODE model:

It allows for an increase in *Foll* over time even if its initial value was 0, i.e. $y_{FOLL}(0)= 0$. In the BDE system, each initial period with a biologically reasonable time course contains at least one period with $Foll=1$, which allows the follicle to be switched ON also later in time. Therefore, this term is not needed in the BDE model.

For finding biologically reasonable values for the delays, one could use either experimental time series data or trajectories from an existing ODE model that has been validated with time series data. Since time series data are not available here, the trajectories of the ODE model are used. In these trajectories, the time differences between the ascents or descents of the various components are monitored and used as initial guess for the delays. These values were adjusted by hand later on until the simulation results fitted the time course of the ODE trajectories. The delay matrix was finally defined as

$$\begin{aligned}\varTheta = \left( \begin{array}{cccccccccc} - &{} - &{}- &{}0.050&{} -&{} -&{}-&{} -&{} 0.050&{} -\\ 0.025&{} - &{} - &{}0.050&{} -&{} -&{} - &{} - &{} - &{} - \\ - &{}0.550 &{}1.000&{} - &{} - &{} 0.100 &{} - &{} 0.550 &{} - &{} - \\ - &{} - &{}0.100&{} -&{} - &{} - &{} - &{} - &{} -&{} - \\ - &{} - &{} - &{}0.100&{} -&{} - &{} - &{} - &{} 0.050 &{} - \\ - &{} - &{}0.050&{} - &{}0.050&{} - &{} - &{} - &{} - &{} - \\ - &{} -&{} -&{} - &{} -&{} -&{} - &{} - &{} - &{} 0.050 \\ - &{} 0.800&{} -&{}0.300&{} - &{}- &{} 0.050 &{} 0.500 &{} - &{} - \\ - &{} - &{} - &{} -&{} -&{} - &{} - &{} 0.025 &{} - &{} - \\ - &{} - &{} - &{} -&{} -&{} - &{} - &{} 0.050 &{} - &{} - \\ \end{array}\right) . \end{aligned}$$

The largest delay is $\theta _{3,3}=1$, which describes the delay of *CL* influencing itself. This delay also defines the cycle length, as it leads to *CL* reproducing its own state after the delay, see also Sect. 3.3.

The trajectory of the ODE model was also used to find a suitable initial period that resulted in a biologically meaningful BDE trajectory. This initial period was then replaced by the values of the BDE simulation after a short non-periodic transient to obtain periodicity right from the beginning of the simulation.

#### Simulation Results

**Fig. 2:**
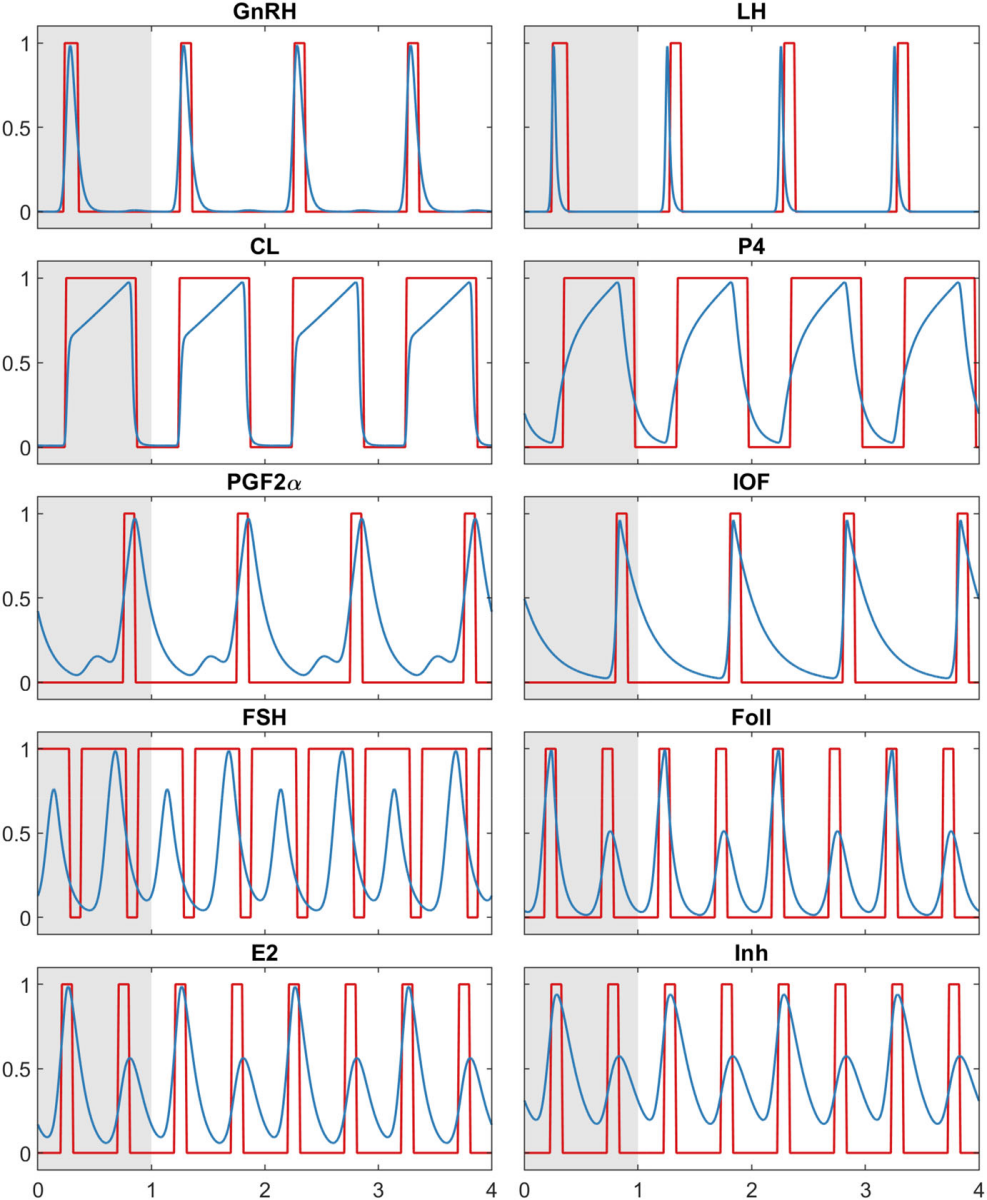
*Model A* (red) and reduced BovCycle (blue), simulation results for all 10 components over a time span of four period lengths. The grey part indicates the initial period in whose range the values of the BDE system were not simulated but entered as initial values for the simulation. The BDE system is periodic right from the beginning of the simulation, with a period length of 1. The simulation results of the reduced BovCycle ODE model were scaled to the same period length for comparability (Color figure online)

The simulation results in Fig. 2 are shown in comparison with the reduced BovCycle (blue lines). It can be seen that *Model A* nicely reproduces the ON and OFF switching of the components in the ODE model, thus providing comparable trajectories.

This is not surprising, since the BDE model was derived from the ODE trajectories and equations. However, the clear consistency of the trajectories also shows that the abstraction is actually able to preserve the qualitative behaviour of the more complex system. Furthermore, the model is capable of switching between cycles with two or three follicular waves by a simple scaling of some delays (see Sect. 3.1.3 for details).

The original ODE BovCycle model can also reproduce experimental data that shows a shift of the ovulation time after administration of $PGF2\alpha$ at certain time points in the cycle (Stötzel et al. 2012). Note, however, that this works only for the original model. With the reduced BovCycle model, it is not possible to conduct this experiment successfully (Stötzel 2014).

When *Model A* is challenged with this experiment, it also fails at this task. This is not unexpected since the model is derived from the reduced ODE model rather than from the original ODE model. The reason for the failure is that some mechanisms that cause the shift are missing in both the reduced ODE model and *Model A*. The shift of ovulation in the original ODE model is based on the following cascade of hormones influencing one another:

$$\begin{aligned} \begin{array}{c} \hbox {Administration of } PGF2\alpha \hbox { inhibits }CL\\ \downarrow \\ \hbox {A smaller }CL\hbox { produces less }P4\\ \downarrow \\ \hbox {Low }P4\hbox { activates }GnRH\hbox { (only if the value of }E2 \hbox { is sufficiently high)}\\ \downarrow \\ GnRH\hbox { activates }LH \hbox {(which is equivalent to ovulation).} \end{array} \end{aligned}$$

This cascade of dependencies leads to an earlier ovulation compared to a cycle without interventions. In *Model A*, the administration does not have the desired effect, hence this experiment fails. The reason for this is that in *Model A*, *CL* depends on *IOF*: When the administration of $PGF2\alpha$ is set to the correct time and causes *IOF* to be switched ON, it actually causes *CL* to be turned OFF. However, this in turn switches *IOF* OFF, which leads to *CL* being activated. The short disturbance of *CL* induced in this way has no influence on the time course of *GnRH*, and therefore cannot shift the cycle.

#### Varying the Number of Follicular Waves

It is described that the bovine estrous cycle mostly has either two or three follicular waves (Adams 1999). The ODE model can also show both behaviours, depending on parameter values (Stötzel et al. 2014b).

In nature, four of the modelled species can show different wave patterns, in most cases two or three follicular waves per cycle, namely *FSH*, *Foll*, *E*2, and *Inh*. To induce this change of the wave pattern in the model simulations, the delays of these species onto themselves (represented by the entries in the lower right $4\times 4$ block in the delay matrix) need to be multiplied by a factor of $\frac{2}{3}$. This factor is due to the fact that the components that previously had two waves in one cycle should now have three waves. The delays are therefore shortened in order to compress the time course of these four components and to produce the desired number of waves.

The resulting delay matrix (changed values in bold text) is then

$$\begin{aligned} \varTheta = \left( \begin{array}{cccccccccc} - &{} - &{}- &{}0.050&{} -&{}- &{}- &{} - &{}0.050 &{} - \\ 0.025&{} - &{} - &{}0.050&{} -&{}- &{} - &{} - &{} - &{} - \\ - &{}0.550 &{}1.000&{} -&{} - &{}0.100&{}- &{}0.550 &{} - &{} - \\ - &{} - &{}0.100&{} -&{}- &{}- &{} - &{}- &{} - &{} - \\ - &{} - &{} - &{}0.100&{} - &{}- &{} - &{} - &{}0.050 &{} - \\ - &{} - &{}0.050&{} - &{}0.050&{}- &{} - &{} - &{} - &{} - \\ - &{} -&{} -&{} - &{} -&{}- &{} - &{} - &{} - &{}\mathbf{0} .\mathbf{033} \\ - &{} 0.800&{} -&{}0.300&{} - &{}- &{} \mathbf{0} .\mathbf{033} &{} \mathbf{0} .\mathbf{333} &{} - &{} - \\ - &{} - &{} - &{} -&{} - &{}- &{} - &{} \mathbf{0} .\mathbf{017} &{} - &{} - \\ - &{} - &{} - &{} -&{} - &{}- &{} - &{} \mathbf{0} .\mathbf{033} &{} - &{} - \\ \end{array}\right) . \end{aligned}$$

**Fig. 3:**
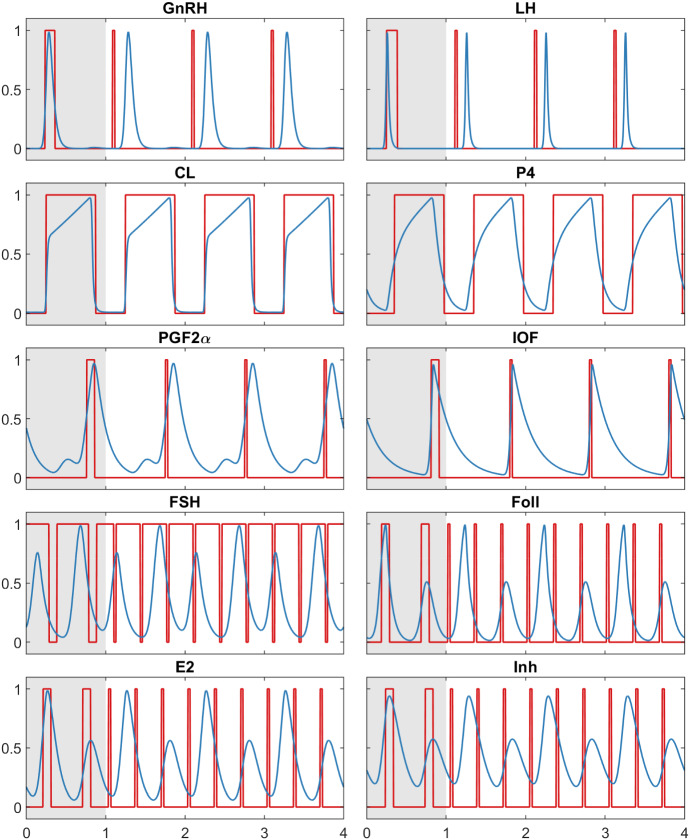
Simulation result for all ten components of *Model A* (red) with a change in five delays over a time span of four period lengths, compared to the reduced BovCycle model (blue). *Model A* can be switched by simply changing the delays so that there are three follicular waves per cycle instead of two. This is visible here for the model components *FSH*, *Foll*, *E*2 and *Inh*. The other six components have in both cases one peak per cycle (Color figure online)

The simulation results for this experiment are shown in Fig. 3. The initial period was the same as for the simulation with two follicular waves as can be seen in the first part of the plots. As soon as the numerical solution of the BDE model starts (at $t=1$), the four components that should switch to three waves show the desired behaviour. The other six species remain peaking once per cycle. It can be seen that after the switching, the peak times no longer match those of the ODE for all components. It is clear that the four components that switch from two to three peaks now have different peak times. Among the components that peak once per cycle, the peak of *GnRH*, *LH*, and $PGF2\alpha$ is shifted to an earlier time. This effect could not be prevented by adjusting the delays.

Even though *Model A* can reflect different wave patterns, its inability of capturing the administration of $PGF2\alpha$ led us to the development of a second BDE model.

### Model B

Treatment with $PGF2\alpha$ is frequently conducted in cows to synchronise the estrous cycles in a group of cows to reduce the costs for artificial insemination. The biological background and underlying mechanisms are described in detail by Stötzel et al. (2012). It is shown there that the BovCycle model is able to reproduce the shift of ovulation following administration of $PGF2\alpha$ at a specific point in time.

To obtain a BDE model that can reproduce this behaviour, the logical equations of *Model B* are not only based on the equations derived from Stötzel et al. (2015), but they are complemented and improved by physiological knowledge.

#### Equations and Delays

All logical functions are the same as for *Model A*, except the functions for *Foll* and *CL*, which look as follows:

$$\begin{aligned} CL&= \lnot PGF2\alpha \wedge (LH \vee CL) \\ Foll&=\lnot LH \wedge FSH \end{aligned}$$

The luteolytic action of $PGF2\alpha$, i.e. its negative effect on *CL*, is well-known (Wenzinger and Bleul 2012), as explained in Sect. 3.2.3. Therefore, it is reasonable to implement this mechanism directly into the equation of *CL*. Since the *CL* evolves during ovulation, which is indicated by a peak of *LH*, the positive influence of *LH* on *CL* is needed to initiate the growth of *CL*. To ensure that $CL=1$ is maintained during the luteal phase, *CL* has a positive feedback on itself. It keeps itself alive as long as $PGF2\alpha$ is not ON.

As the name suggests, the hormone *FSH* is stimulating the follicles and therefore has a positive impact on *Foll*. *LH* is active shortly before ovulation, when the dominant follicle ovulates, and therefore leads to a significant drop in the size of all follicles, which explains the negative influence of *LH* on *Foll*.

The delay matrix for *Model B* is

$$\begin{aligned} \varTheta = \frac{1}{4.5}\left( \begin{array}{cccccccccc} - &{} - &{}- &{}0.75&{} -&{}-&{}- &{} -&{}0.75&{} -\\ 0.75&{} - &{} - &{}0.75&{} -&{}-&{} - &{} - &{} - &{} - \\ - &{}0.75 &{}0.75&{} -&{} 4.5 &{}-&{}-&{}-&{} - &{} - \\ - &{} - &{}1.5&{} -&{}- &{}-&{} - &{}- &{} - &{} - \\ - &{} - &{} - &{}0.75&{} - &{}-&{} - &{} - &{}0.75&{} - \\ - &{} - &{}1.5&{} - &{}0.75&{}-&{} - &{} - &{} - &{} - \\ - &{} -&{} -&{} - &{} -&{}-&{} - &{} - &{} - &{}3 \\ - &{}0.75&{} -&{}-&{} - &{}-&{} 1.5 &{}-&{} - &{} - \\ - &{} - &{} - &{} -&{} - &{}-&{} - &{}0.75&{} - &{} - \\ - &{} - &{} - &{} -&{} - &{}-&{} - &{}1.5&{} - &{} - \\ \end{array}\right) . \end{aligned}$$

*Model B* should have a physiologically reasonable cycle length of 21 days, but in contrast to *Model A* it should not have the cycle length fixed by a delay with the value of the cycle length. The factor in front of the matrix is for rescaling purpose, so that the largest delay is 1. Before rescaling, the delays of the successive switchings add up to the desired cycle length of 21 days, see also Sect. 3.3 and Fig. 6. *IOF* does not influence any other component in *Model B*, unlike in *Model A*, where it occurs in the logical equation for *CL*. Therefore, *IOF* is redundant in this model. However, *IOF* was not removed from *Model B* to make the simulation results comparable with those of *Model A*.

#### Simulation Results

**Fig. 4:**
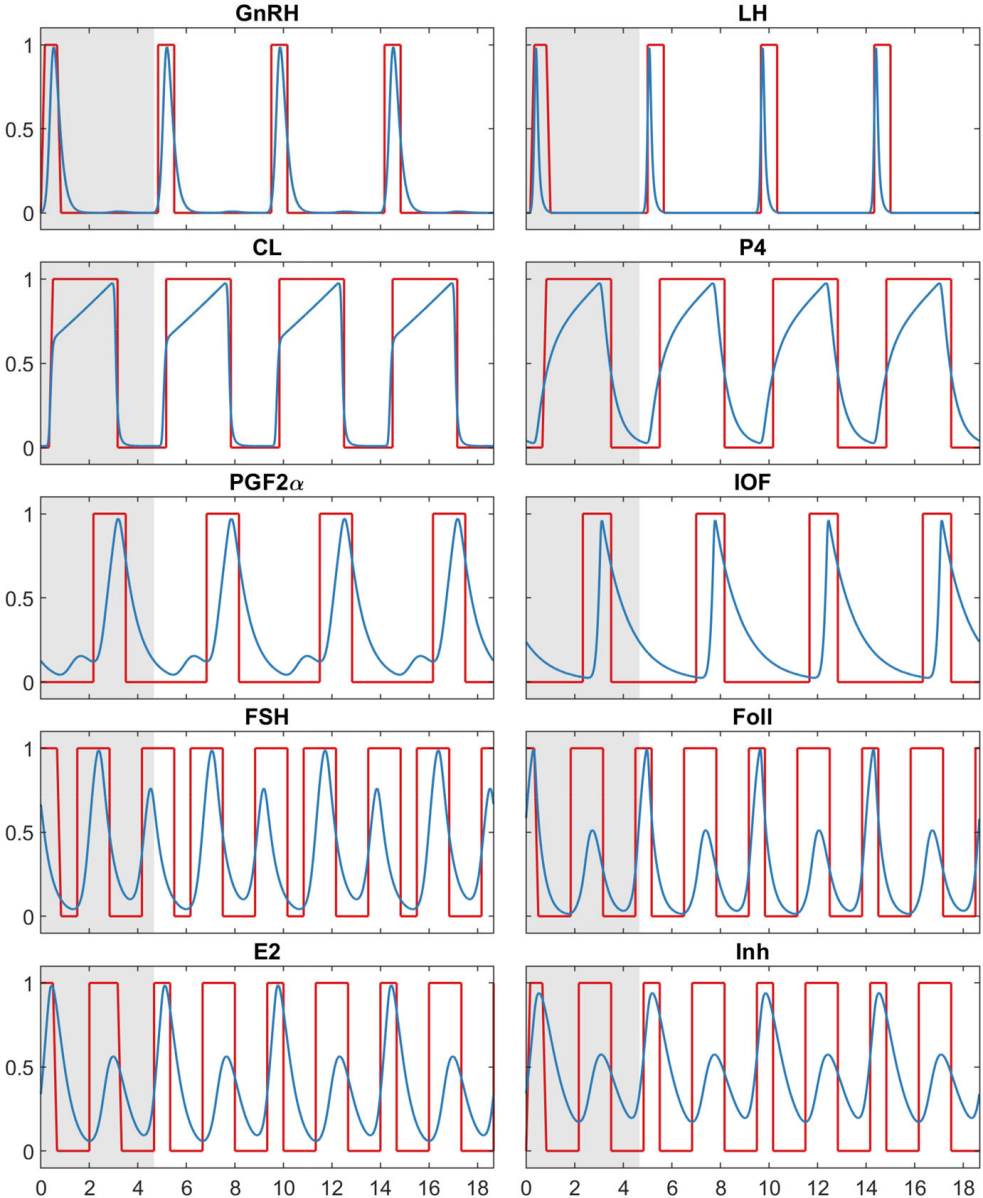
Simulation results for *Model B* (red) compared to the reduced BovCycle model (blue) for all ten model components. The grey highlighted part indicates the initial period. The period length of *Model B* is $4.{\overline{6}}$. The simulation results of the reduced BovCycle were scaled to the same period length (Color figure online)

The simulation results for all components are shown in Fig. 4. Initially, the same initial period as in *Model A* was used. After the simulation became periodic following a short transient, this periodic part was used as initial period so that this simulation is also periodic from the beginning.

Again, the simulation results are shown in comparison with the reduced BovCycle model (blue lines). *Model B* also reproduces the behaviour of the ODE model well. The period length of *Model B* is $4.{\overline{6}}$. The reason for this is that this BDE model was designed to have a physiologically reasonable period length of 21 days (see Fig. 6). The largest delay in the resulting BDE model is 4.5. Therefore, the delay matrix is multiplied by $\frac{1}{4.5}$ to scale the model in a way that the largest delay is 1. This scaling of the delay matrix leads to a period length of $\frac{21}{4.5}=4.{\overline{6}}$.

#### Administration of $PGF2\alpha$

Prostaglandin F2$\alpha$ ($PGF2\alpha$) is commonly used in estrous synchronisation protocols, making use of its luteolytic action. It causes luteolysis in cows, which means regression of the corpus luteum, and therefore ends the luteal phase, so that a new estrous cycle can start (Okuda et al. 2002).

**Fig. 5:**
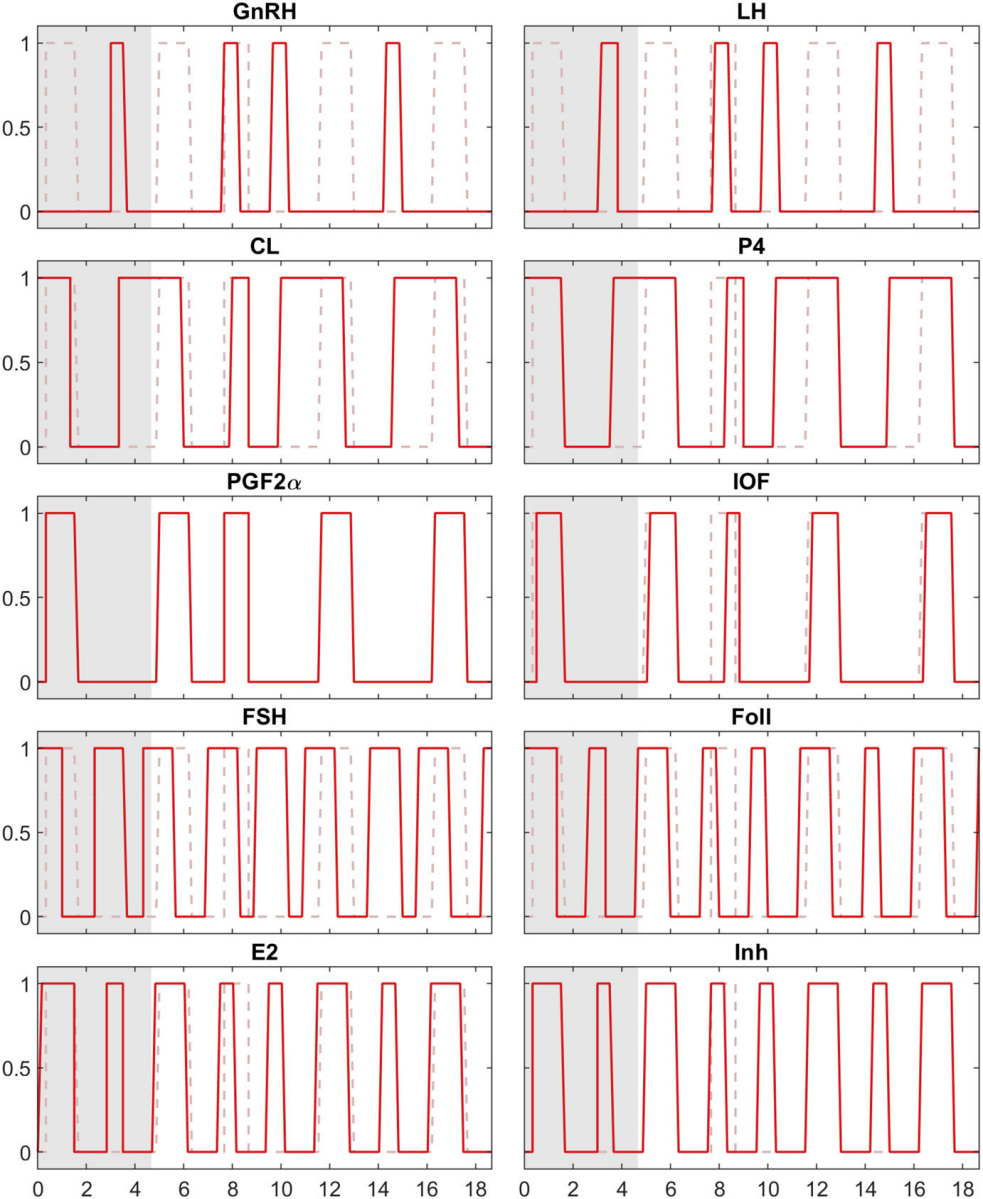
Simulation results for *Model B* with $PGF2\alpha$ administration at time $t=7.67$ for a duration of 1 day. The red solid lines show the simulation results for all ten species. The dashed light red line in all subplots shows the time course of $PGF2\alpha$ to illustrate the influence of the administration on the other species. It can be seen how *CL* is switched OFF shortly after the beginning of the administration of $PGF2\alpha$. As in the other figures, the grey highlighted part indicates the initial period

In Fig. 5, the results of a numerical simulation of $PGF2\alpha$ administration are presented. The value of the species $PGF2\alpha$ was manually set to 1 during the simulation for a duration of 1 day at time $t=7.67$. All other species are simulated without direct manual intervention. The simulation results show that *LH* is switching on earlier than in the simulation without $PGF2\alpha$ administration. Then, the cycle continues so that a synchronisation could be simulated successfully. The cycle length after synchronisation is the same as before due to the fixed values of the delays.

This experiment does not work for administration of $PGF2\alpha$ at any time in the cycle. The time point $t=7.67$ is chosen to be approximately in the middle of the first time window in which certain conditions are satisfied. In the BDE model, the condition for a successful shift of the cycle is that a period of $CL=1$ overlaps with the administration period (shifted by the corresponding delay, which is in this model $\theta _{3,5}=1$) so that *CL* can be switched OFF. Another condition is that *E*2 is at value 1 at the right time, since *GnRH* can be activated by switching OFF *P*4 only if $E2=1$.

This observation coincides with experimental results on administration of $PGF2\alpha$ at different stages in the cycle. In Wenzinger and Bleul (2012), a $PGF2\alpha$ analogue is administered on days 3 and 5 after ovulation. The authors report that ovulation can not be induced by the drug administered on day 3, but in five out of eight cows on day 5, indicating that $PGF2\alpha$ has no effect when administered too early in the cycle. This is consistent with the behaviour of the BDE model as the value of *CL* is 0 at earlier administration time points so that no shift in ovulation would occur. Thus, this result serves as an additional validation of *Model B*.

### Trajectories and Periodicity

The observations in this section serve for model validation because they confirm that the models reproduce biological knowledge about the cycle. They also establish a context between the abstract model states and biological concepts in the different phases of the cycle.

The normal range for the length of the bovine estrous cycle is 18–24 days (Forde et al. 2011). In contrast to *Model B*, *Model A* is not designed to match a given period length, but to reproduce the simulation results of the reduced ODE BovCycle model. The largest delay is $\theta _{3,3}=1$, which is also the cycle length. The absolute value of the cycle length has no particular significance at this point, since the length is derived from the largest delay ($\theta _{3,3}$), which is scaled to 1 and represents the delay of *CL* influencing itself. The corpus luteum renews itself after expiration of one cycle.

**Fig. 6:**
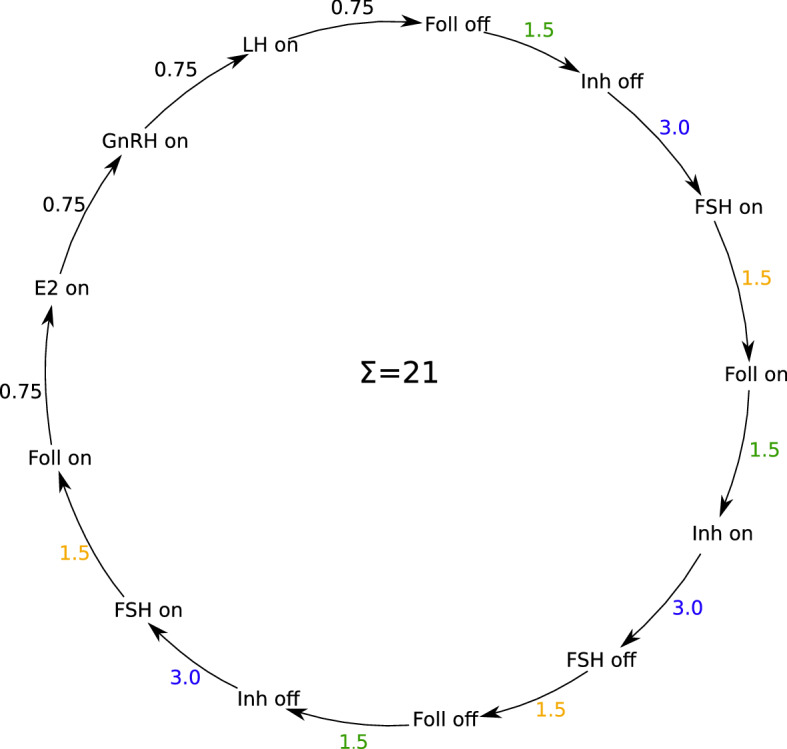
Delays and cycle length for *Model B*. It can be seen that the delays of the components that switch one after the other add up to the desired cycle length of 21 days. Numbers with the same colours indicate that the same delay applies. It should be noted that the black numbers represent different delays. It should also be noted that not all switching components are represented in the circle, but only those that directly influence each other and ultimately cause the *LH* peak and therefore ovulation

*Model B* is designed to have the same cycle length of 21 days as the BovCycle model. This cycle length is obtained by setting the influential delays to values which add up to the desired length of 21 days. This is shown in Fig. 6. Not all model components are part of the cycle, but all (except *IOF*) are necessary so that the components can pass through the cycle. In particular, the influence of the other components is necessary at the time point where *GnRH* is switched on. This can only happen because *P*4 is switched OFF at the appropriate time.

The number of times at which components are turned ON or OFF is 28 for both models as well as for the trajectory of the Boolean model described by Stötzel et al. (2015). In the Boolean model, no two switchings can happen at the same time, as the model was implemented with asynchronous updates. In both BDE models, some components switch at the same time. Therefore, counting the states passed through during one cycle results in 23 states for *Model A* and 21 states for *Model B*.

**Fig. 7:**
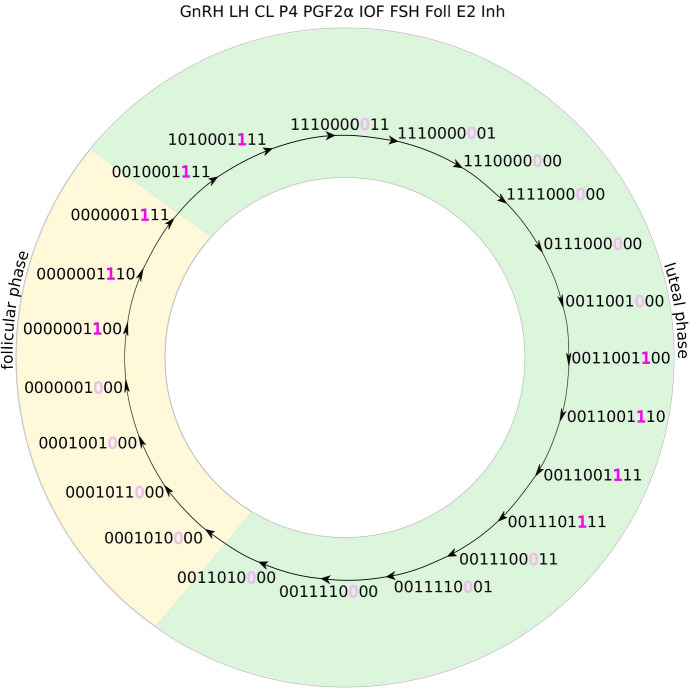
This image shows the trajectory of *Model A*. The ten numbers of each state are the values of the ten components of the model in the order as stated above the circle. The pink/light pink colour of the eighth component (*Foll*) indicates the two follicular waves per cycle. The green background is for the luteal phase (here defined as states with $CL=1$, represented by the third component) and the yellow background for the follicular phase (Color figure online)

In Fig. 7, the trajectory of *Model A* with two waves is shown together with colour markings for the follicular phase (yellow background) and the luteal phase (green background). The two follicular waves are marked by the colour differences of the eighth digit of the states, which represents the component *Foll*. In this case, the luteal phase is defined as the phase in which *CL* has the value 1. The figure shows 23 different states. Since in several cases not only one but up to three components switch at the same time, the total number of ON and OFF switches of components per cycle is 28. We provide the same figure for a cycle with three follicular waves as Online Resource 3.

**Fig. 8:**
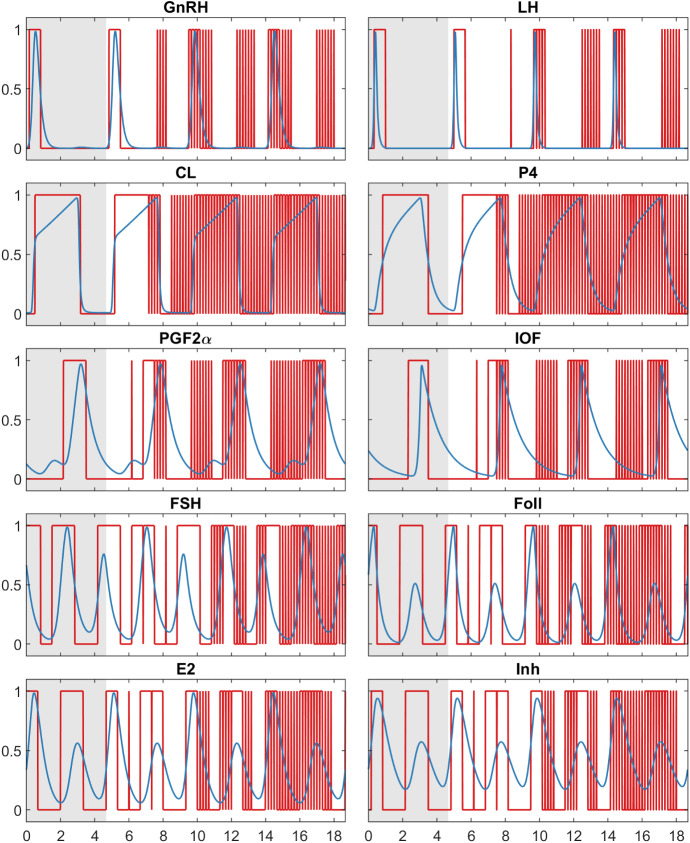
This figure shows the simulation result for *Model B* with one changed delay: $\theta _{2,4}= 0.165= \frac{0.75}{4.5}\cdot 0.99$. The completely different behaviour of the model for a small change of only one parameter illustrates the very high sensitivity of the model towards changes of the delays

### Sensitivity

The herein constructed BDE models are very sensitive with respect to changes in some of the model parameters, i.e. delays and initial periods. That means, even very small changes in the input parameters can lead to a completely different periodic behaviour of the BDE system. At this point, no global investigation of the sensitivity is carried out. Instead, we demonstrate with the following example how a small change of only one delay significantly disturbs the behaviour of the simulation and leads to a biologically unreasonable result. For this purpose, the delay of *P*4 influencing *LH* in *Model B* is changed by 1%. The original value of $\theta _{2,4}= \frac{0.75}{4.5}$ is replaced by $\theta _{2,4}= 0.165= \frac{0.75}{4.5}\cdot 0.99$. The results of the simulation are shown in Fig. 8. It is clearly visible that all components have a strongly increased number of jump points. This result cannot be interpreted biologically. This high sensitivity with regard to single delays can also be observed for other delays, not only in *Model B*, but also in *Model A*, where $\theta _{3,3}$ is an example for a highly sensitive parameter.

As a consequence of this high sensitivity, an automated optimisation of the highly sensitive parameters with respect to time series data is extremely difficult, which is a major disadvantage. For this reason, the delays and initial periods were fitted manually during the creation of both *Model A* and *Model B*. In addition, the number of possible jump points during the computation of the solution is determined by the least common multiple of the denominators of all delays in $\varTheta$. Small perturbations of the delays can therefore lead to a strong increase in this value and therefore in the number of possible jump points. In these cases, the computing time is extremely prolonged, which makes automatic optimisation even more challenging. However, the high sensitivity does not apply to all delay parameters. For less sensitive parameters, automatic optimisation might be possible. A high sensitivity towards specific parameters also shows up in the ODE model BovCycle, where for example the cycle length is very sensitive towards changes in the parameter for the blood volume. It should be noted that the disadvantage of high sensitivity cannot be concluded for BDEs in general, but only for the two models presented here.

## Conclusion and Future Work

Two BDE models of the bovine estrous cycle have been created and presented in this article. The simulation results and equations of the ODE model BovCycle were used as a guideline, in addition to known physiological findings for the bovine estrous cycle.

The trajectories of both models match well with the ODE trajectories, but neither reproduces the exact trajectory of the Boolean model in Stötzel et al. (2015). However, the switching sequences resulting from the two BDE models are just two out of several possible discrete representations for the signalling cascade that drives the bovine estrous cycle. The reason for the existence of multiple discrete representations of the switching order is the fact that the components are continuous in vivo and that some hormones can have different thresholds for different sites of action. Therefore, it is not possible to uniquely determine at which value in the continuous space a component in the discrete space must be ON or OFF.

In comparison with ODE models or with time series data, both of which are not discrete, it is difficult to decide on the quality of a discrete trajectory like the one produced by a BDE model. To make a direct comparison, one would have to discretise the continuous ODE trajectories or time series data. The result of this discretisation, however, highly depends on the choice of thresholds. Therefore, it is a more appropriate method to use qualitative properties and experiments for validation.

*Model A* can be switched between two and three follicular waves by a simple scaling of some particular delays. With *Model B*, one can successfully simulate a shift of the estrous cycle following the administration of $PGF2\alpha$ at specific time points that fulfil certain conditions on the development of the *CL*. This result coincides with experimental observations by Wenzinger and Bleul (2012), which validates *Model B*. To our knowledge, this is the first time that a BDE model has been used to simulate the effect of drug administration.

Both experiments (switching the number of waves and shifting the cycle) are only possible with one model and not with the other. With *Model B*, it is not possible to conduct the switching to three follicular waves in the same way as with *Model A*. In *Model A*, the delays of four components were multiplied by $\frac{2}{3}$ to switch from a cycle with two follicular waves to a cycle with three follicular waves. If the same scaling of the delays is applied to *Model B*, one can observe a reduced cycle length, while the number of follicular waves per cycle remains two. The reason for this is that the delays determine the cycle length. For example, as shown in Fig. 6, the delay of *GnRH* influencing *LH* is part of the summed up cycle length of 21 days. If this delay is reduced, the cycle length is shortened at the same time. The structure of *Model B* does not allow for a change of the number of follicular waves by simply scaling the delays of the involved species.

It is known that more than 95% of all cycles in cows have either 2 or 3 follicular waves (Adams and Singh 2014). Therefore, it is an interesting observation that *Model A* can reflect both wave behaviours. However, switching between 2 or 3 follicular waves is not an experiment that can be directly induced in real life. Therefore, it does not argue against the quality of *Model B* that it cannot reproduce this switching. Synchronisation by administration of $PGF2\alpha$, on the contrary, is in vivo a very frequently performed protocol. The inability of *Model A* to simulate these protocols is inherited from the ODE model it was constructed from.

An approach to construct a BDE model by fitting it to time series data derived from a given ODE model is presented by Doherty et al. (2017). Based on the work by Akman et al. (2012), Doherty et al. (2017) use a given ODE model and a BDE model for the same system (a simple circadian clock model) and optimise the parameters of the BDE model by discretising the trajectories of the ODE model and using them as artificially generated time series data. They test different approaches for the optimisation of combinations of three different types of parameters: (i) gate configuration parameters of the logical functions (which, for example, determine if a term stands for the logical identity or for the logical negation), (ii) thresholds for the discretisation of the ODE trajectories, and (iii) delays. The methods by Doherty et al. (2017) are much less manual compared to the herein presented approach. Once the optimisation method is chosen, one can almost automatically generate a BDE from an ODE, provided that the logical functions for the BDE are given in a very basic form.

However, applying the method by Doherty et al. (2017) to the BovCycle model appears to be challenging, as the number of parameters in the models for the bovine estrous cycle is much larger, containing ten thresholds, 17 and 20 delays for *Model A* and *Model B*, respectively, and a yet unknown number of gate parameters (to be determined by constructing a more abstract version of the logical equations, using gate parameters instead of logical connectives). In contrast, the model by Doherty et al. (2017) contains only two thresholds, three delays, and three gate parameters. Nevertheless, the approach of fitting BDEs to data seems promising since the number of parameters is smaller than in corresponding ODE models, which could facilitate the reverse-engineering of biochemical networks. However, a potential problem in estimating the parameters in the bovine estrous cycle BDE models is the high sensitivity of solutions to changes in the parameters. Since the number of possible jump points can increase strongly upon changes in the delays, the computing time for an optimisation can become very long. An idea to continue the work presented here would be to find a reproducible formalism for automatically transferring an ODE model into a BDE model, similar to what was done by Stötzel et al. (2015) to convert an ODE model into a Boolean one. One would have to define a threshold for each species, and then find a method to extract the delays from the discretised trajectories, possibly making use of the thresholds within the Hill functions that occur very frequently in the ODE model. For defining the logical functions, the procedure described by Stötzel et al. (2015) could be used as a guideline.

All in all, some steps were carried out manually during the development of the BDE models. For future work, it would be desirable to perform these steps more automatically. The difficulty here is that the computing effort is very high due to the size of the models and the number of parameters. Nevertheless, this step should be taken in the future to make the procedure applicable to other models and to ensure reproducibility.

We also want to extend the model by the dependence of *FSH* on *GnRH*, which is included neither in *Model A* nor in *Model B*. This extension of the model will hopefully allow to successfully simulate the biological impact of the pathological condition of persisting follicles or *CL*. Experience has shown, however, that the delays need to be adjusted when changing the logical functions, so this extension involves a certain effort. Overall, it seems most promising to first carry out future work related to the automatisation of modelling and to the use of optimisation algorithms, since this could result in considerable time savings compared to manual adaptation and extension of the model.

To summarise, our results support the role of BDEs as original framework. As our motivation for modelling is to construct a model that is able to reproduce the behaviour of a real object as well as possible, we conclude with the recommendation to construct a BDE model based on biological knowledge rather than deriving it from an existing ODE model. Furthermore, a comparative analysis of several models of the same system can also lead to a gain in knowledge, since deficits can be discovered in the individual models that would not become apparent in the analysis of a single model. Our conclusion on the usefulness of BDEs for modelling periodic hormonal systems is that the difficulty of parameter estimation and the very high sensitivity towards some of the delays are major disadvantages compared to modelling with ODEs. However, much less detailed information is needed to construct a BDE model, so that a rather rough model can be generated much more easily if only qualitative knowledge of the underlying system is available and no time series data. We therefore see the modelling with BDEs as a useful complementary tool in the process of gaining mechanistic insight into complex biological systems.

## Supplementary Information

Below is the link to the electronic supplementary material.

Supplementary material 1 (pdf 79 KB)

Supplementary material 2 (pdf 1221 KB)

Supplementary material 3 (pdf 115 KB)

Supplementary material 4 (txt 6 KB)

Supplementary material 5 (txt 6 KB)

Supplementary material 6 (txt 3 KB)

Supplementary material 7 (txt 4 KB)

## Footnotes

1 However, there is the possibility of integrating time delays into Boolean models by adding additional auxiliary variables that can cause a delay, as shown by Hinkelmann and Laubenbacher (2011).

2 For example, in estrous synchronisation protocols a certain percentage of cows, depending on the protocol, are not in estrous at the expected time.

## Declarations

### Code Availability

Within this work, all BDEs were solved using an implementation in Matlab R2018b, which is provided as Online Resource 4.
