# Supplementary material for: Modelling Oscillatory Patterns in the Bovine Estrous Cycle with Boolean Delay Equations
Source: Bull Math Biol. 2021 Nov 2;83(12):121. doi: 10.1007/s11538-021-00942-z (PMC8563642; doi:10.1007/s11538-021-00942-z)
Supplement: Supplementary file 1 — Supplementary material 1 (pdf 79 KB) [file 11538_2021_942_MOESM1_ESM.pdf]

Modelling oscillatory patterns in the bovine estrous cycle with Boolean Delay Equations, Bulletin of Mathematical Biology

Mascha Berg, Julia Plöntzke, Heike Siebert, Susanna Röblitz
Zuse Institute Berlin. Germany, berg@zib.de

Ordinary differential equations of the reduced BovCycle model as published in Stötzel, C., Apri, M., Röblitz, S. (2014). *A reduced ODE model of the bovine estrous cycle.*

$$\frac{d}{dt}y_{GnRH}(t) = m^{GnRH}_{P4,E2} \cdot H^{+}(y_{E2}(t); T^{GnRH}_{E2}, n^{GnRH}_{E2}) \cdot H^{-}(y_{P4}(t); T^{GnRH}_{P4}, n^{GnRH}_{P4}) - c_{GnRH} \cdot y_{GnRH}(t)$$

$$\frac{d}{dt}y_{FSH}(t) = m^{FSH}_{Inh} \cdot H^{-}(y_{Inh}(t); T^{FSH}_{Inh}, n^{FSH}_{Inh}) - c_{FSH} \cdot y_{FSH}(t)$$

$$\frac{d}{dt}y_{LH}(t) = m^{LH}_{GnRH,P4} \cdot H^{-}(y_{P4}(t); T^{LH}_{P4}, n^{LH}_{P4}) \cdot H^{+}(y_{GnRH}(t); T^{LH}_{GnRH}, n^{LH}_{GnRH}) - c_{LH} \cdot y_{LH}(t)$$

$$\begin{aligned}\frac{d}{dt}y_{Foll}(t) = \; & m^{Foll}_{FSH} \cdot H^{+}(y_{FSH}(t); T^{Foll}_{FSH}, n^{Foll}_{FSH}) \cdot (1 + H^{+}(y_{Foll}(t); T^{Foll}_{Foll}, n^{Foll}_{Foll})) \\ & - (m^{Foll}_{P4} \cdot H^{+}(y_{P4}(t); T^{Foll}_{P4}, n^{Foll}_{P4}) + m^{Ovul}_{LH} \cdot H^{+}(y_{LH}(t); T^{Ovul}_{LH}, n^{Ovul}_{LH})) \cdot y_{Foll}(t)\end{aligned}$$

$$\frac{d}{dt}y_{CL}(t) = SF \cdot m^{Ovul}_{LH} \cdot H^{+}(y_{LH}(t); T^{Ovul}_{LH}, n^{Ovul}_{LH}) \cdot y_{Foll}(t) + m^{CL}_{CL} \cdot H^{+}(y_{CL}(t); T^{CL}_{CL}, n^{CL}_{CL}) - m^{CL}_{IOF} \cdot H^{+}(y_{IOF}(t); T^{CL}_{IOF}, n^{CL}_{IOF}) \cdot y_{CL}(t)$$

$$\frac{d}{dt}y_{P4}(t) = k^{P4}_{CL} \cdot y_{CL}(t) - c_{P4} \cdot y_{P4}(t)$$

$$\frac{d}{dt}y_{E2}(t) = k^{E2}_{Foll} \cdot y_{Foll}(t) - c_{E2} \cdot y_{E2}(t)$$

$$\frac{d}{dt}y_{Inh}(t) = k^{Inh}_{Foll} \cdot y_{Foll}(t) - c_{Inh} \cdot y_{Inh}(t)$$

$$\frac{d}{dt}y_{PGF}(t) = H^{+}(y_{E2}(t); T^{PGF}_{E2}, n^{PGF}_{E2}) \cdot H^{+}(y_{P4}(t); T^{PGF}_{P4}, n^{PGF}_{P4}) - c_{PGF} \cdot y_{PGF}(t)$$

$$\frac{d}{dt}y_{IOF}(t) = m^{IOF}_{PGF,CL} \cdot H^{+}(y_{PGF}(t); T^{IOF}_{PGF}, n^{IOF}_{PGF}) \cdot H^{+}(y_{CL}(t); T^{IOF}_{CL}, n^{IOF}_{CL}) - c_{IOF} \cdot y_{IOF}(t)$$

$H^{+/-}(y; T, n)$ is a positive/negative Hill function for the component y with threshold T and exponent n.

The values of the model parameters $m^{GnRH}_{P4,E2}$, m^{FSH}_{Inh}, m^{Ovul}_{LH}, $m^{LH}_{GnRH,P4}$, m^{Foll}_{FSH}, m^{CL}_{IOF}, m^{CL}_{CL}, $m^{IOF}_{PGF,CL}$, m^{Foll}_{P4}, k^{P4}_{CL}, k^{E2}_{Foll}, k^{Inh}_{Foll}, c_{GnRH}, c_{FSH}, c_{LH}, c_{P4}, c_{E2}, c_{Inh}, c_{PGF}, c_{IOF}, SF and the Hill thresholds can be found in the original publication.

The Hill exponents are set as $n^{GnRH}_{E2} = n^{FSH}_{Inh} = n^{Foll}_{P4} = n^{CL}_{IOF} = n^{PGF}_{P4} = n^{IOF}_{PGF} = n^{IOF}_{CL} = 5$, while the other Hill exponents have the value 2.
