# Supplementary figures and images for: Modelling Oscillatory Patterns in the Bovine Estrous Cycle with Boolean Delay Equations
Source: Bull Math Biol. 2021 Nov 2;83(12):121. doi: 10.1007/s11538-021-00942-z (PMC8563642; doi:10.1007/s11538-021-00942-z)

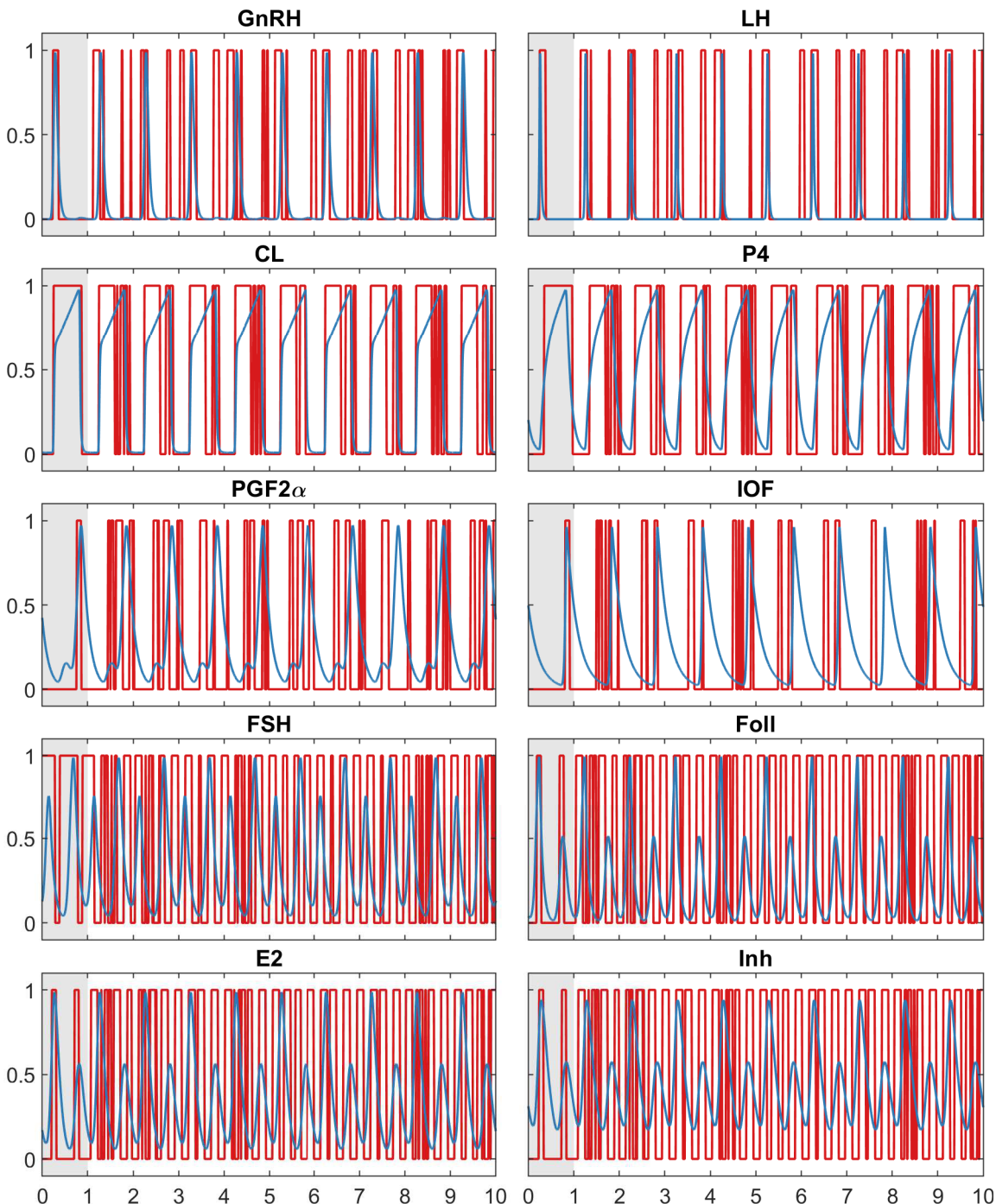

Supplement: Supplementary file 2 — Supplementary material 2 (pdf 1221 KB) [file 11538_2021_942_MOESM2_ESM.pdf]

GnRH LH CL P4 PGF2 $\alpha$  IOF FSH Foll E2 Inh

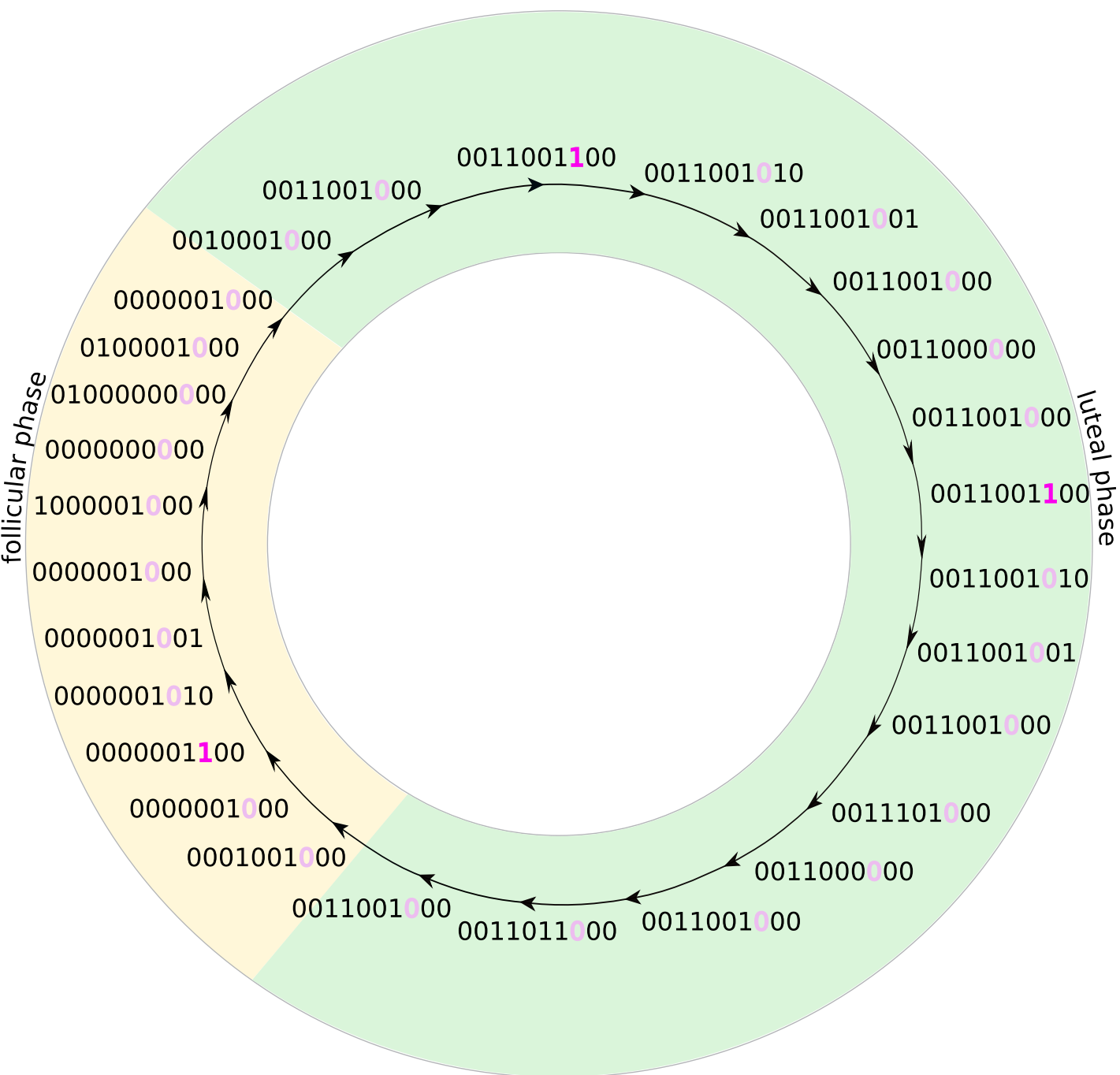

Supplement: Supplementary file 3 — Supplementary material 3 (pdf 115 KB) [file 11538_2021_942_MOESM3_ESM.pdf]
